# Supplementary material for: FSC-certified forest management benefits large mammals compared to non-FSC
Source: Nature. 2024 Apr 10;628(8008):563–8. doi: 10.1038/s41586-024-07257-8 (PMC11023928; doi:10.1038/s41586-024-07257-8)
Supplement: Supplementary file 1 — Supplementary Tables 1, 2 and 4. [file 41586_2024_7257_MOESM1_ESM.pdf]

nature portfolio

https://doi.org/10.1038/s41586-024-07257-8

Supplementary information

FSC-certified forest management benefits large mammals compared to non-FSC

In the format provided by the authors and unedited

Supplementary Table 1. Principles and criteria of the FSC International Standard related to environmental impacts. There is no hierarchy between the Principles or between Criteria. They share equal status, validity and authority, and apply jointly and severally at the level of the individual Management Unit. For a complete overview of all principles and criteria, as well as verifiers and indicators, see the FSC international standard, 2015.

1) Compliance with Laws - The Organization shall comply with all applicable laws, regulations and nationally- ratified international treaties, conventions and agreements.

1.4) The Organization shall develop and implement measures, and/or shall engage with regulatory agencies, to systematically protect the Management Unit from unauthorized or illegal resource use, settlement and other illegal activities.

1.5) The Organization shall comply with the applicable national laws, local laws, ratified international conventions and obligatory codes of practice, relating to the transportation and trade of forest products within and from the Management Unit, and/or up to the point of first sale.

3) Indigenous Peoples' Rights - The Organization shall identify and uphold Indigenous Peoples' legal and customary rights of ownership, use and management of land, territories and resources affected by management activities.

3.1) The Organization shall identify the Indigenous Peoples that exist within the Management Unit or are affected by management activities. The Organization shall then, through engagement with these Indigenous Peoples, identify their rights of tenure, their rights of access to and use of forest resources and ecosystem services, their customary rights and legal rights and obligations, that apply within the Management Unit. The Organization shall also identify areas where these rights are contested.

3.2) The Organization shall recognize and uphold the legal and customary rights of Indigenous Peoples to maintain control over management activities within or related to the Management Unit to the extent necessary to protect their rights, resources and lands and territories. Delegation by Indigenous Peoples of control over management activities to third parties requires Free, Prior and Informed Consent.

3.5) The Organization, through engagement with Indigenous Peoples, shall identify sites which are of special cultural, ecological, economic, religious or spiritual significance and for which these Indigenous Peoples hold legal or customary rights. These sites shall be recognized by The Organization and their management, and/or protection shall be agreed through engagement with these Indigenous Peoples.

4) Community Relations - The Organization shall contribute to maintaining or enhancing the social and economic wellbeing of local communities.

4.1) The Organization shall identify the local communities that exist within the Management Unit and those that are affected by management activities. The Organization shall then, through engagement with these local communities, identify their rights of tenure, their rights of access to and use of forest resources and ecosystem services, their customary rights and legal rights and obligations, that apply within the Management Unit.

4.2) The Organization shall recognize and uphold the legal and customary rights of local communities to maintain control over management activities within or related to the Management Unit to the extent necessary to protect their rights, resources, lands and territories. Delegation by traditional peoples of control over management activities to third parties requires Free, Prior and Informed Consent.

4.5) The Organization, through engagement with local communities, shall take action to identify, avoid and mitigate significant negative social, environmental and economic impacts of its management activities on affected communities. The action taken shall be proportionate to the scale, intensity and risk of those activities and negative impacts.

5) Benefits from the Forest - The Organization shall efficiently manage the range of multiple products and services of the Management Unit to maintain or enhance long term economic viability and the range of environmental and social benefits.

5.1) The Organization shall identify, produce, or enable the production of, diversified benefits and/or products, based on the range of resources and ecosystem services existing in the Management Unit in order to strengthen and diversify the local economy proportionate to the scale and intensity of management activities.

5.3) The Organization shall demonstrate that the positive and negative externalities of operation are included in the management plan.

6) Environmental Values and Impacts - The Organization shall maintain, conserve and/or restore ecosystem services and environmental values of the Management Unit, and shall avoid, repair or mitigate negative environmental impacts.

6.4) The Organization shall protect rare species and threatened species and their habitats in the Management Unit through conservation zones, protection areas, connectivity and/or (where necessary) other direct measures for their survival and viability. These measures shall be proportionate to the scale, intensity and risk of management activities and to the conservation status and ecological requirements of the rare and threatened species. The Organization shall take into account the geographic range and ecological requirements of rare and threatened species beyond the boundary of the Management Unit, when determining the measures to be taken inside the Management Unit.

6.6) The Organization shall effectively maintain the continued existence of naturally occurring native species and genotypes, and prevent losses of biological diversity, especially through habitat management in the Management Unit. The Organization shall demonstrate that effective measures are in place to manage and control hunting, fishing, trapping and collecting.

7) Management Planning - The Organization shall have a management plan consistent with its policies and objectives and proportionate to scale, intensity and risks of its management activities. The management plan shall be implemented and kept up to date based on monitoring information in order to promote adaptive management. The associated planning and procedural documentation shall be sufficient to guide staff, inform affected stakeholders and interested stakeholders and to justify management decisions.

7.1) The Organization shall, proportionate to scale, intensity and risk of its management activities, set policies (visions and values) and objectives for management, which are environmentally sound, socially beneficial and economically viable. Summaries of these policies and objectives shall be incorporated into the management plan, and publicized.

7.4) The Organization shall update and revise periodically the management planning and procedural documentation to incorporate the results of monitoring and evaluation, stakeholder engagement or new scientific and technical information, as well as to respond to changing environmental, social and economic circumstances.

8) Monitoring and Assessment - The Organization shall demonstrate that, progress towards achieving the management objectives, the impacts of management activities and the condition of the Management Unit, are monitored and evaluated proportionate to the scale, intensity and risk of management activities, in order to implement adaptive management.

8.1) The Organization shall monitor the implementation of its management plan, including its policies and objectives, its progress with the activities planned, and the achievement of its verifiable targets.

8.2) The Organization shall monitor and evaluate the environmental and social impacts of the activities carried out in the Management Unit, and changes in its environmental condition.

8.3) The Organization shall analyze the results of monitoring and evaluation and feed the outcomes of this analysis back into the planning process.

9) High Conservation Values - The Organization shall maintain and/or enhance the High Conservation Values in the Management Unit through applying the precautionary approach.

9.1) The Organization, through engagement with affected stakeholders, interested stakeholders and other means and sources, shall assess and record the presence and status of the following High Conservation Values in the Management Unit, proportionate to the scale, intensity and risk of impacts of management activities, and likelihood of the occurrence of the High Conservation Values:

HCV 1 - Species diversity. Concentrations of biological diversity including endemic species, and rare, threatened or endangered species, that are significant at global, regional or national levels.

HCV 2 - Landscape-level ecosystems and mosaics. Intact forest landscapes and large landscape-level ecosystems and ecosystem mosaics that are significant at global, regional or national levels, and that contain viable populations of the great majority of the naturally occurring species in natural patterns of distribution and abundance.

HCV 3 - Ecosystems and habitats. Rare, threatened, or endangered ecosystems, habitats or refugia.

HCV 4 - Critical ecosystem services. Basic ecosystem services in critical situations, including protection of water catchments and control of erosion of vulnerable soils and slopes.

HCV 5 - Community needs. Sites and resources fundamental for satisfying the basic necessities of local communities or Indigenous Peoples (for livelihoods, health, nutrition, water, etc.), identified through engagement with these communities or Indigenous Peoples.

HCV 6 - Cultural values. Sites, resources, habitats and landscapes of global or national cultural, archaeological or historical significance, and/or of critical cultural, ecological, economic or religious/sacred importance for the traditional cultures of local communities or Indigenous Peoples, identified through engagement with these local communities or Indigenous Peoples.

10) Implementation of Management Activities - Management activities conducted by or for The Organization for the Management Unit shall be selected and implemented consistent with The Organization's economic, environmental and social policies and objectives and in compliance with the Principles and Criteria collectively.

10.10) The Organization shall manage infrastructural development, transport activities and silviculture so that water resources and soils are protected, and disturbance of and damage to rare and threatened species, habitats, ecosystems and landscape values are prevented, mitigated and/or repaired.

Supplementary Table 2. National criteria and indicators of the FSC standards regarding hunting of Gabon and The Republic of Congo. Comparable indicators and criteria are highlighted with numbers in superscript. For a complete overview of all principles and criteria, as well as verifiers and indicators, see the FSC National Forest Stewardship Standard of The Gabonese Republic (2020) and The Republic of Congo (2020).

**Gabon**	**Republic of Congo**
**Indicator 1.4.1**[1]<br>Measures are implemented to provide protection from unauthorized or illegal harvesting, hunting, fishing, trapping, collecting, settlement and other unauthorized activities, notably:<br>1) Security gates on main forest roads and/ or control of access to high-risk areas;<br>2) Closure of temporal roads after harvesting;<br>3) Surveillance patrols on forest road to detect and report to the forest administration, any illegal access to the forest; and<br>4) Designation of personnel and resources to rapidly detect and monitor illegal activities.	**Indicator 1.4.1**[1]<br>Measures are implemented to provide protection from unauthorized or illegal harvesting, hunting, fishing, trapping, collecting, settlement and other unauthorized activities, notably:<br>1) Security gates on main forest roads and/ or control of access to high-risk areas;<br>2) Closure of temporal roads after harvesting;<br>3) Surveillance patrols on forest road to detect and prevent illegal access to the forest; and<br>4) Designation of personnel and resources to rapidly detect and monitor illegal activities during the exercise of legal user rights.
**Indicator 6.4.4**[2&7]<br>The Organization puts in place mechanisms to ensure that:<br>1) Hunting, trapping and fishing are prevented in accordance with applicable regulations;<br>2) Applicable national and/or international regulations on protection, hunting, fishing and trade in animal species or parts (trophies) are known and complied with;<br>3) There are internal regulations prohibiting and penalizing illegal hunting, fishing and collecting within the MU, and the transport and trade in bush meat and firearms in the concession-holder's vehicles;<br>4) There are internal procedures to control illegal hunting, fishing and collecting practices within the Management Unit.	
**Criterion 6.6**[3]<br>The Organization shall effectively maintain the continued existence of naturally occurring native species and genotypes, and prevent losses of biological diversity, especially through habitat management in the Management Unit. The Organization shall demonstrate that effective measures are in place to manage and control hunting, fishing, trapping and collecting.	**Criterion 6.6**[3]<br>The Organization shall effectively maintain the continued existence of naturally occurring native species and genotypes, and prevent losses of biological diversity, especially through habitat management in the Management Unit. The Organization shall demonstrate that effective measures are in place to manage and control hunting, fishing, trapping and collecting.
**Indicator 6.6.4**[4]<br>Effective measures are taken to manage and control hunting, fishing, trapping and collecting activities to ensure that naturally occurring native species, their diversity within species and their natural distribution are maintained.	**Indicator 6.6.4**[4]<br>Effective measures are taken, and personnel appointed, to manage and control hunting, fishing, trapping and collecting activities to ensure that the diversity and natural distribution of native species is maintained.
**Indicator 6.6.5**[5]<br>A system of regular and punctual controls is implemented to ensure hunting policies are respected.	**Indicator 6.6.5**[2]<br>Mechanisms for wildlife protection are in place: Applicable national and/or international regulations on protection, hunting and trade in animal species or parts (trophies) shall be known and complied with.
**Indicator 6.6.6**[6]<br>Effective mitigation measures are in place to ensure that workers do not increase the practice of hunting, trapping or collecting of bush meat or wild fish.	**Indicator 6.6.6**[7]<br>There is an internal regulation banning and punishing the transportation of and trade in bush meat, firearms, munitions and hunters in the vehicles belonging to The Organization and its sub-contractors, and governing the keeping of firearms on The Organization's premises. This regulation shall be known, disseminated and complied with.
	**Indicator 6.6.7**[5]<br>A system of regular and punctual controls to ensure hunting policies are respected is implemented.
	**Indicator 6.6.8**[6]<br>Effective mitigation measures are in place to regulate the practices of hunting, trapping or collecting of bush meat or wild fish by the workers of The Organization.

Supplementary Table 4. List of tested models to assess potential covariate influence of geographic and camera trap site covariates. Linear mixed-effects models for mammal encounter rates ('rai') were specified with concession pairs ('pair'), concessions ('sitename') and cameras ('camera') as random effects, whereby cameras were nested within concessions within concession pairs, in a multi-level random effect structure. Geographic covariates included elevation ('elevation'), distance to roads ('dist_roads'), distance to rivers ('dist_rivers'), distance to settlements ('dist_settlements') and distance to protected areas ('dist_protected areas'). All geographic covariates were also tested with quadratic terms. Camera trap site covariates included the availability of water within 50 m ('water') or fruiting trees within 30 m ('fruit_trees'), visibility and the presence of trails or paths ('type_of_site'). Relative importance of models was tested using a model-selection approach based on minimization of Bayesian Information Criterion (BIC) values, reported alongside the number of parameters (npar) and model structures. Models are sorted by ascending BIC in separate analyses for all species combined, for body mass classes, taxonomic groups, and IUCN Red List categories.

Model	npar	BIC
All species – Geographic covariates		
log_rai ~ certification + elevation + (1|pair) + (1|concession) + (1|camera)	7	1086.4
log_rai ~ certification + dist_rivers + (1|pair) + (1|concession) + (1|camera)	7	1087.9
log_rai ~ certification + exp(elevation) + (1|pair) + (1|concession) + (1|camera)	7	1088.3
log_rai ~ certification + exp(dist_rivers) + (1|pair) + (1|concession) + (1|camera)	7	1089.8
log_rai ~ certification + (1|pair) + (1|concession) + (1|camera)	6	1090.7
log_rai ~ certification + exp(dist_roads) + (1|pair) + (1|concession) + (1|camera)	7	1091.2
log_rai ~ certification + dist_roads + (1|pair) + (1|concession) + (1|camera)	7	1093.5
log_rai ~ certification + dist_protected_areas + (1|pair) + (1|concession) + (1|camera)	7	1093.9
log_rai ~ certification + exp(dist_protected_areas) + (1|pair) + (1|concession) + (1|camera)	7	1094.9
log_rai ~ certification + dist_settlements + (1|pair) + (1|concession) + (1|camera)	7	1095.3
log_rai ~ certification + exp(dist_settlements) + (1|pair) + (1|concession) + (1|camera)	7	1096.3
log_rai ~ certification + elevation + dist_roads + dist_rivers + dist_settlements + dist_protected_areas + (1|pair) + (1|concession) + (1|camera)	11	1099.6
log_rai ~ certification + exp(elevation) + exp(dist_roads) + exp(dist_rivers) + exp(dist_settlements) + exp(dist_protected_areas) + (1|pair) + (1|concession) + (1|camera)	11	1106.8
All species – Camera trap site covariates		
log_rai ~ certification + (1|pair) + (1|concession) + (1|camera)	6	1085.8
log_rai ~ certification + water + (1|pair) + (1|concession) + (1|camera)	7	1088.5
log_rai ~ certification + fruit_trees + (1|pair) + (1|concession) + (1|camera)	7	1091.8
log_rai ~ certification + visibility + (1|pair) + (1|concession) + (1|camera)	8	1097.2
log_rai ~ certification + type_of_site + (1|pair) + (1|concession) + (1|camera)	9	1100.4
log_rai ~ certification + water + visibility + fruit_trees + type_of_site + (1|pair) + (1|concession) + (1|camera)	13	1120
Weight classes – Geographic covariates		
log_rai ~ certification * weightclass + (weightclass|pair) + (1|concession) + (1|camera)	28	6980.2
log_rai ~ certification * weightclass + elevation + (weightclass|pair) + (1|concession) + (1|camera)	29	6981.2
log_rai ~ certification * weightclass + dist_settlements + (weightclass|pair) + (1|concession) + (1|camera)	29	6982.5
log_rai ~ certification * weightclass + exp(dist_protected_areas) + (weightclass|pair) + (1|concession) + (1|camera)	29	6983.3
log_rai ~ certification * weightclass + dist_protected_areas + (weightclass|pair) + (1|concession) + (1|camera)	29	6983.6
log_rai ~ certification * weightclass + exp(dist_roads) + (weightclass|pair) + (1|concession) + (1|camera)	29	6983.7
log_rai ~ certification * weightclass + dist_roads + (weightclass|pair) + (1|concession) + (1|camera)	29	6983.7
log_rai ~ certification * weightclass + exp(elevation) + (weightclass|pair) + (1|concession) + (1|camera)	29	6983.9
log_rai ~ certification * weightclass + dist_rivers + (weightclass|pair) + (1|concession) + (1|camera)	29	6984.6
log_rai ~ certification * weightclass + exp(dist_rivers) + (weightclass|pair) + (1|concession) + (1|camera)	29	6984.9
log_rai ~ certification * weightclass + exp(dist_settlements) + (weightclass|pair) + (1|concession) + (1|camera)	29	6985.2
log_rai ~ certification * weightclass + elevation + dist_roads + dist_rivers + dist_settlements + dist_protected_areas + (weightclass|pair) + (1|concession) + (1|camera)	33	6999
log_rai ~ certification * weightclass + exp(elevation) + exp(dist_roads) + exp(dist_rivers) + exp(dist_settlements) + exp(dist_protected_areas) + (weightclass|pair) + (1|concession) + (1|camera)	33	7008.4
Weight classes – Camera trap site covariates		
log_rai ~ certification * weightclass + (weightclass|pair) + (1|concession) + (1|camera)	28	6937.1
log_rai ~ certification * weightclass + water + (weightclass|pair) + (1|concession) + (1|camera)	29	6940.5
log_rai ~ certification * weightclass + fruit_trees + (weightclass|pair) + (1|concession) + (1|camera)	29	6944.6
log_rai ~ certification * weightclass + visibility + (weightclass|pair) + (1|concession) + (1|camera)	30	6950.2
log_rai ~ certification * weightclass + type_of_site + (weightclass|pair) + (1|concession) + (1|camera)	31	6956.7
log_rai ~ certification * weightclass + water + visibility + fruit_trees + type_of_site + (weightclass|pair) + (1|concession) + (1|camera)	35	6980.7
Taxonomic groups – Geographic covariates		
log_rai ~ certification * taxonomy + (taxonomy|pair) + (1|concession) + (1|camera)	36	7846.6
log_rai ~ certification * taxonomy + elevation + (taxonomy|pair) + (1|concession) + (1|camera)	37	7848.3
log_rai ~ certification * taxonomy + exp(elevation) + (taxonomy|pair) + (1|concession) + (1|camera)	37	7851.1
log_rai ~ certification * taxonomy + dist_settlements + (taxonomy|pair) + (1|concession) + (1|camera)	37	7852.2
log_rai ~ certification * taxonomy + exp(dist_protected_areas) + (taxonomy|pair) + (1|concession) + (1|camera)	37	7852.6
log_rai ~ certification * taxonomy + dist_protected_areas + (taxonomy|pair) + (1|concession) + (1|camera)	37	7852.6
log_rai ~ certification * taxonomy + exp(dist_roads) + (taxonomy|pair) + (1|concession) + (1|camera)	37	7853.1
log_rai ~ certification * taxonomy + exp(dist_settlements) + (taxonomy|pair) + (1|concession) + (1|camera)	37	7853.5
log_rai ~ certification * taxonomy + dist_rivers + (taxonomy|pair) + (1|concession) + (1|camera)	37	7853.7
log_rai ~ certification * taxonomy + exp(dist_rivers) + (taxonomy|pair) + (1|concession) + (1|camera)	37	7854.1
log_rai ~ certification * taxonomy + dist_roads + (taxonomy|pair) + (1|concession) + (1|camera)	37	7854.7

log_rai ~ certification * taxonomy + elevation + dist_roads + dist_rivers + dist_settlements + dist_protected_areas + (taxonomy|pair) + (1|concession) + (1|camera)	41	7863.5
log_rai ~ certification * taxonomy + exp(elevation) + exp(dist_roads) + exp(dist_rivers) + exp(dist_settlements) + exp(dist_protected_areas) + (taxonomy|pair) + (1|concession) + (1|camera)	41	7877
Taxonomic groups – Camera trap site covariates		
log_rai ~ certification * taxonomy + (taxonomy|pair) + (1|concession) + (1|camera)	36	7794.5
log_rai ~ certification * taxonomy + water + (taxonomy|pair) + (1|concession) + (1|camera)	37	7799
log_rai ~ certification * taxonomy + fruit_trees + (taxonomy|pair) + (1|concession) + (1|camera)	37	7804.7
log_rai ~ certification * taxonomy + visibility + (taxonomy|pair) + (1|concession) + (1|camera)	38	7807.2
log_rai ~ certification * taxonomy + type_of_site + (taxonomy|pair) + (1|concession) + (1|camera)	39	7815.4
log_rai ~ certification * taxonomy + water + visibility + fruit_trees + type_of_site + (taxonomy|pair) + (1|concession) + (1|camera)	43	7844.1
IUCN Red List categories – Geographic covariates		
log_rai ~ certification * iucn + (iucn|pair) + (1|concession) + (1|camera)	28	6169.5
log_rai ~ certification * iucn + dist_roads + (iucn|pair) + (1|concession) + (1|camera)	29	6173
log_rai ~ certification * iucn + elevation + (iucn|pair) + (1|concession) + (1|camera)	29	6173
log_rai ~ certification * iucn + exp(dist_roads) + (iucn|pair) + (1|concession) + (1|camera)	29	6174.2
log_rai ~ certification * iucn + dist_protected_areas + (iucn|pair) + (1|concession) + (1|camera)	29	6175.7
log_rai ~ certification * iucn + dist_settlements + (iucn|pair) + (1|concession) + (1|camera)	29	6175.7
log_rai ~ certification * iucn + exp(elevation) + (iucn|pair) + (1|concession) + (1|camera)	29	6176.1
log_rai ~ certification * iucn + exp(dist_rivers) + (iucn|pair) + (1|concession) + (1|camera)	29	6176.2
log_rai ~ certification * iucn + dist_rivers + (iucn|pair) + (1|concession) + (1|camera)	29	6176.7
log_rai ~ certification * iucn + exp(dist_protected_areas) + (iucn|pair) + (1|concession) + (1|camera)	29	6177
log_rai ~ certification * iucn + exp(dist_settlements) + (iucn|pair) + (1|concession) + (1|camera)	29	6177.1
log_rai ~ certification * iucn + elevation + dist_roads + dist_rivers + dist_settlements + dist_protected_areas + (iucn|pair) + (1|concession) + (1|camera)	33	6194.4
log_rai ~ certification * iucn + exp(elevation) + exp(dist_roads) + exp(dist_rivers) + exp(dist_settlements) + exp(dist_protected_areas) + (iucn|pair) + (1|concession) + (1|camera)	33	6200.6
IUCN Red List categories – Camera trap site covariates		
log_rai ~ certification * iucn + (iucn|pair) + (1|concession) + (1|camera)	28	6131.7
log_rai ~ certification * iucn + water + (iucn|pair) + (1|concession) + (1|camera)	29	6134.5
log_rai ~ certification * iucn + fruit_trees + (iucn|pair) + (1|concession) + (1|camera)	29	6138
log_rai ~ certification * iucn + visibility + (iucn|pair) + (1|concession) + (1|camera)	30	6142.6
log_rai ~ certification * iucn + type_of_site + (iucn|pair) + (1|concession) + (1|camera)	31	6147.9
log_rai ~ certification * iucn + water + visibility + fruit_trees + type_of_site + (iucn|pair) + (1|concession) + (1|camera)	35	6170.1
