# Supplementary material for: FSC-certified forest management benefits large mammals compared to non-FSC
Source: Nature. 2024 Apr 10;628(8008):563–8. doi: 10.1038/s41586-024-07257-8 (PMC11023928; doi:10.1038/s41586-024-07257-8)
Supplement: Supplementary file 2 — Reporting Summary [file 41586_2024_7257_MOESM2_ESM.pdf]

nature portfolio

Corresponding author(s): J.A. Zwerts

Last updated by author(s): Feb 9, 2024

Reporting Summary

Nature Portfolio wishes to improve the reproducibility of the work that we publish. This form provides structure for consistency and transparency in reporting. For further information on Nature Portfolio policies, see our Editorial Policies and the Editorial Policy Checklist.

Statistics

For all statistical analyses, confirm that the following items are present in the figure legend, table legend, main text, or Methods section.

n/a	Confirmed	
☐	☒	The exact sample size (*n*) for each experimental group/condition, given as a discrete number and unit of measurement
☐	☒	A statement on whether measurements were taken from distinct samples or whether the same sample was measured repeatedly
☐	☒	The statistical test(s) used AND whether they are one- or two-sided<br>*Only common tests should be described solely by name; describe more complex techniques in the Methods section.*
☐	☒	A description of all covariates tested
☐	☒	A description of any assumptions or corrections, such as tests of normality and adjustment for multiple comparisons
☐	☒	A full description of the statistical parameters including central tendency (e.g. means) or other basic estimates (e.g. regression coefficient) AND variation (e.g. standard deviation) or associated estimates of uncertainty (e.g. confidence intervals)
☐	☒	For null hypothesis testing, the test statistic (e.g. *F*, *t*, *r*) with confidence intervals, effect sizes, degrees of freedom and *P* value noted<br>*Give P values as exact values whenever suitable.*
☒	☐	For Bayesian analysis, information on the choice of priors and Markov chain Monte Carlo settings
☐	☒	For hierarchical and complex designs, identification of the appropriate level for tests and full reporting of outcomes
☒	☐	Estimates of effect sizes (e.g. Cohen's *d*, Pearson's *r*), indicating how they were calculated

Our web collection on statistics for biologists contains articles on many of the points above.

Software and code

Policy information about availability of computer code

Data collection	No software was used for data collection, all photos were annotated in the program Wild.ID.
Data analysis	Statistical analyses were performed in R version 4.2.2. R code for statistical analyses and data tables is available in the Zenodo repository under https://doi.org/10.5281/zenodo.10061155.

For manuscripts utilizing custom algorithms or software that are central to the research but not yet described in published literature, software must be made available to editors and reviewers. We strongly encourage code deposition in a community repository (e.g. GitHub). See the Nature Portfolio guidelines for submitting code & software for further information.

Data

Policy information about availability of data

All manuscripts must include a data availability statement. This statement should provide the following information, where applicable:

- Accession codes, unique identifiers, or web links for publicly available datasets
- A description of any restrictions on data availability
- For clinical datasets or third party data, please ensure that the statement adheres to our policy

The data that support the findings of this study is available in the Zenodo repository under https://doi.org/10.5281/zenodo.10061155

Research involving human participants, their data, or biological material

Policy information about studies with human participants or human data. See also policy information about sex, gender (identity/presentation), and sexual orientation and race, ethnicity and racism.

Reporting on sex and gender	N/A
Reporting on race, ethnicity, or other socially relevant groupings	N/A
Population characteristics	N/A
Recruitment	N/A
Ethics oversight	N/A

Note that full information on the approval of the study protocol must also be provided in the manuscript.

Field-specific reporting

Please select the one below that is the best fit for your research. If you are not sure, read the appropriate sections before making your selection.

☐ Life sciences ☐ Behavioural & social sciences ☒ Ecological, evolutionary & environmental sciences

For a reference copy of the document with all sections, see nature.com/documents/nr-reporting-summary-flat.pdf

Ecological, evolutionary & environmental sciences study design

All studies must disclose on these points even when the disclosure is negative.

Study description	We used camera traps to assess whether FSC certification can mitigate the negative effects of timber extraction on wildlife by studying the encounter rate of a broad range of mammal species across multiple sites.
Research sample	We compared small to large-sized mammal observations across seven paired FSC-certified and non-FSC concessions in Gabon and the Republic of Congo. Gabon and the Republic of Congo lie within Western Equatorial Africa (WEA), and we included all companies that were FSC-certified between 2018 and 2021 in this region, with the exception of one which refused to allow access. WEA is particularly suitable for these analyses, as its forests are reasonably intact and logging concessions are embedded in a matrix of contiguous forest, which are therefore mostly devoid of influences other than the effects of logging and hunting. Wild meat hunting is pervasive in WEA. Logging increases hunting pressure by increased access (logging roads) and by the arrival of people working in the concessions in once-remote forests. By ensuring close spatial pairing of the FSC-certified and non-FSC concessions we reduced the influence of regional landscape heterogeneity.
Sampling strategy	We set up arrays of camera traps from 2018 to 2021 in 14 logging concessions owned by 11 different companies (5 FSC and 6 non-FSC) in Gabon and the Republic of Congo. We included all companies that were FSC-certified between 2018 and 2021 in this region, with the exception of one which refused to allow access. Seven FSC-certified concessions were each paired to the closest non-FSC concession that was similar in terms of terrain and forest type. All concessions are situated in a matrix of connected forests. Within each pair of concessions, camera traps (Bushnell Trophy Cam HD for pairs 1 - 6 and Browning 2018 Spec Ops Advantage for pair 7) were deployed simultaneously to account for seasonal differences, for two to three months. There was one exception where Covid restrictions obliged the cameras to remain in place for longer. Camera trap grid locations within each pair of concessions were chosen based on similarity between potential drivers of mammal abundance, including distance to settlements, roads, rivers, protected areas, elevation and time since logging (2-10 years before our study), although some camera grids overlapped older logging blocks.
Data collection	Camera traps were set out in systematic, one-kilometre spaced grids with a random start point. Upon reaching the predetermined GPS locations, the first potential installation location was used where cameras had at least 4 metres of visibility. This ensured that each grid was representative of environmental heterogeneity: i.e. not specifically targeting nor ignoring trails or other landscape elements that could influence detection. The one-kilometre inter-camera distance exceeds most species' home range sizes to avoid spatial autocorrelation. Neither were species expected to migrate within the sampling duration of the study. Between 28 to 36 cameras were deployed in each concession, totalling up to 474 camera traps, distributed over 474 km2. Cameras were installed at a height of 30 cm to enable observations of mammals of all sizes while ensuring that each camera had at least four metres visibility in front of it. Cameras were programmed to take bursts of three photos to maximize the chance of detection and to take a photo every 12 hours for correct calculation of active days in the event of a defect before the end of the deployment period. For each camera, we recorded whether there was an elephant path skidder trail, small wildlife trail, or none of the above within each camera's field of view. We also visually estimated forest visibility (0-10m / 11-20m / >20m), slope (0-5° / 5-20° / >20°), presence of fruiting trees within 30 m and presence of small water courses within 50 m. When approaching each predefined camera point, we counted cartridges, snares and hunting camps from 500 m before the camera up to its location. Various field teams were employed in different sites and hence there may be some influence interobserver bias of hunting observations between sites.
Timing and spatial scale	We set up arrays of camera traps from 2018 to 2021. Camera traps were deployed simultaneously to account for seasonal

Timing and spatial scale	differences, for two to three months. There was one exception where Covid restrictions obliged the cameras to remain in place for longer.
Data exclusions	No data was excluded from the analysis.
Reproducibility	All camera trap data is available and can be reanalyzed to ensure the reproducibility.
Randomization	Camera trap grid locations within each pair of concessions were chosen based on similarity between potential drivers of mammal abundance, including distance to settlements, roads, rivers, protected areas, elevation and time since logging (2-10 years before our study), although some camera grids overlapped older logging blocks. Camera traps were set out in systematic, one-kilometre spaced grids with a random start point. Upon reaching the predetermined GPS locations, the first potential installation location was used where cameras had at least 4 metres of visibility. This ensured that each grid was representative of environmental heterogeneity: i.e. not specifically targeting nor ignoring trails or other landscape elements that could influence detection. The one-kilometre inter-camera distance exceeds most species' home range sizes to avoid spatial autocorrelation. Neither were species expected to migrate within the sampling duration of the study. Between 28 to 36 cameras were deployed in each concession, totalling up to 474 camera traps, distributed over 474 km2. Cameras were installed at a height of 30 cm to enable observations of mammals of all sizes while ensuring that each camera had at least four metres visibility in front of it. Cameras were programmed to take bursts of three photos to maximize the chance of detection and to take a photo every 12 hours for correct calculation of active days in the event of a defect before the end of the deployment period. For each camera, we recorded whether there was an elephant path skidder trail, small wildlife trail, or none of the above within each camera's field of view. We also visually estimated forest visibility (0-10m / 11-20m / >20m), slope (0-5° / 5-20° / >20°), presence of fruiting trees within 30 m and presence of small water courses within 50 m. When approaching each predefined camera point, we counted cartridges, snares and hunting camps from 500 m before the camera up to its location. Various field teams were employed in different sites and hence there may be some influence interobserver bias of hunting observations between sites.
Blinding	Blinding was not relevant for our study as all mammal observations are linked to locations.

Did the study involve field work? ☒ Yes ☐ No

Field work, collection and transport

Field conditions	Within each pair of concessions, camera traps (Bushnell Trophy Cam HD for pairs 1 - 6 and Browning 2018 Spec Ops Advantage for pair 7) were deployed simultaneously to account for seasonal differences, for two to three months. There was one exception where Covid restrictions obliged the cameras to remain in place for longer. Camera trap grid locations within each pair of concessions were chosen based on similarity between potential drivers of mammal abundance, including distance to settlements, roads, rivers, protected areas, elevation and time since logging (2-10 years before our study), although some camera grids overlapped older logging blocks.
Location	We set up arrays of camera traps from 2018 to 2021 in 14 logging concessions owned by 11 different companies (5 FSC and 6 non-FSC) in Gabon and the Republic of Congo.
Access & import/export	We gained permission from all participating logging companies to access their concessions.
Disturbance	Paths were made in the forest to be able to install the camera traps. Tropical forests are highly resilient such small disturbances as plants regrow quickly.

Reporting for specific materials, systems and methods

We require information from authors about some types of materials, experimental systems and methods used in many studies. Here, indicate whether each material, system or method listed is relevant to your study. If you are not sure if a list item applies to your research, read the appropriate section before selecting a response.

Materials & experimental systems

n/a	Involved in the study	
☒	☐	Antibodies
☒	☐	Eukaryotic cell lines
☒	☐	Palaeontology and archaeology
☐	☒	Animals and other organisms
☒	☐	Clinical data
☒	☐	Dual use research of concern
☒	☐	Plants

Methods

n/a	Involved in the study	
☒	☐	ChIP-seq
☒	☐	Flow cytometry
☒	☐	MRI-based neuroimaging

Animals and other research organisms

Policy information about studies involving animals; ARRIVE guidelines recommended for reporting animal research, and Sex and Gender in Research

Laboratory animals	The study did not involve laboratory animals.
Wild animals	We confirm that no wild animals were harmed in any way as animals were only observed using camera traps.
Reporting on sex	The study did not involve sex-based data or analysis.
Field-collected samples	The study did not involve samples collected from the field.
Ethics oversight	No ethical approval or guidance was required because animals were only remotely observed using camera traps.

Note that full information on the approval of the study protocol must also be provided in the manuscript.
